# RNA-Seq analysis of the multipartite genome of *Rhizobium etli* CE3 shows different replicon contributions under heat and saline shock

**DOI:** 10.1186/1471-2164-15-770

**Published:** 2014-09-08

**Authors:** Gamaliel López-Leal, Maria Luisa Tabche, Santiago Castillo-Ramírez, Alfredo Mendoza-Vargas, Miguel A Ramírez-Romero, Guillermo Dávila

**Affiliations:** Programa de Genómica Evolutiva, Centro de Ciencias Genómicas, Universidad Nacional Autónoma de México, Apartado Postal 565-A, Cuernavaca, Morelos C.P 62210, México; Departamento de Microbiología Molecular, Instituto de Biotecnología, Universidad Nacional Autónoma de México, Cuernavaca, Morelos C.P 62120, México; Instituto Nacional de Medicina Genómica, Secretaría de Salud, Periférico Sur No. 4809, México, DF, 14610 México; Oncomedic. Cuauhtémoc 84A, Col, Torrielo Guerra, México, DF, C.P. 14050, México

## Abstract

**Background:**

*Regulation* of transcription is essential for any organism and *Rhizobium etli* (a multi-replicon, nitrogen-fixing symbiotic bacterium) is no exception. This bacterium is commonly found in the rhizosphere (free-living) or inside of root-nodules of the common bean (*Phaseolus vulgaris*) in a symbiotic relationship. Abiotic stresses, such as high soil temperatures and salinity, compromise the genetic stability of *R. etli* and therefore its symbiotic interaction with *P. vulgaris*. However, it is still unclear which genes are up- or down-regulated to cope with these stress conditions. The aim of this study was to identify the genes and non-coding RNAs (ncRNAs) that are differentially expressed under heat and saline shock, as well as the promoter regions of the up-regulated loci.

**Results:**

Analysing the heat and saline shock responses of *R. etli* CE3 through RNA-Seq, we identified 756 and 392 differentially expressed genes, respectively, and 106 were up-regulated under both conditions. Notably, the set of genes over-expressed under either condition was preferentially encoded on plasmids, although this observation was more significant for the heat shock response. In contrast, during either saline shock or heat shock, the down-regulated genes were principally chromosomally encoded. Our functional analysis shows that genes encoding chaperone proteins were up-regulated during the heat shock response, whereas genes involved in the metabolism of compatible solutes were up-regulated following saline shock. Furthermore, we identified thirteen and nine ncRNAs that were differentially expressed under heat and saline shock, respectively, as well as eleven ncRNAs that had not been previously identified. Finally, using an *in silico* analysis, we studied the promoter motifs in all of the non-coding regions associated with the genes and ncRNAs up-regulated under both conditions.

**Conclusions:**

Our data suggest that the replicon contribution is different for different stress responses and that the heat shock response is more complex than the saline shock response. In general, this work exemplifies how strategies that not only consider differentially regulated genes but also regulatory elements of the stress response provide a more comprehensive view of bacterial gene regulation.

**Electronic supplementary material:**

The online version of this article (doi:10.1186/1471-2164-15-770) contains supplementary material, which is available to authorized users.

## Background

In eubacteria, a primary strategy for gene regulation is the modulation of transcriptional initiation [1–3]. This is typically controlled by an RNA polymerase, along with its sigma factors, and can be modified (positively or negatively) by transcription factors (TFs) [3, 4]. The number of sigma factors and TFs encoded in a given genome varies across bacterial species [5]. Usually, bacteria with large genomes have numerous sigma factors and TFs [6], suggesting a positive correlation between genome size and the content of transcriptional regulators. This observation also applies to the bacterial lifestyle, as large genome-sized species usually dominate in environments where resources are scarce and conditions fluctuate, such as in soil [7, 8]. The use of specific sigma factors and numerous TFs ensures a very specific promoter recognition and highly gene-specific transcriptional control [9–11].

Additionally, small RNA transcripts (typically 50–350 nt), which are not translated into proteins, have recently been identified as key elements that regulate the bacterial stress response [12], pathogenicity and other cellular processes, for example; chromosomal replication, cell division [13], RNA processing [14] and protein stability [15]. These small transcripts are a heterologous group of molecules that can act by base-pairing to mRNAs to modify translation efficiency and the stability of the mRNA (via degradation) [16] or by binding to proteins to modify their activities. In plant symbiotic bacteria, several computational predictions and experimental studies have been conducted to identify and characterize these elements [17, 18]. However, despite those efforts to identify novel ncRNAs, the characterization and identification of ncRNA targets is still a rather unexplored subject [19].

*Rhizobium etli* is a soil bacterium that can associate with the roots of the common bean (*P. vulgaris*) [20]. These bacteria are able to establish a symbiotic relationship with legumes; in such a relationship, the formation of root nodules is induced and the *R. etli* in these nodules differentiates into nitrogen-fixing bacteroide [20]. These bacteroides convert dinitrogen into ammonium, which the plant uses as a nitrogen source. Similar to the genomes of soil-living bacteria, *R. etli* shows high levels of genome plasticity and genome redundancy, which is reflected in the number of sigma factors. In this regard, *R. etli* encodes twenty-three sigma factors, a housekeeping gene σ^70^ (*sigA*), two σ^54^ (*rpoN*), two σ^32^ (*rpoH*) and 18 extracellular factor (ECF) genes. This sigma factor redundancy is also present in other Rhizobia; for instance *Bradyrhizobium japonicum* has 26 sigma factors, *Mesorhizobium loti* encodes 25 and *Sinorhizobium meliloti* contains 16 sigma factors [21–24]. To the contrary, very little is known about the ncRNAs in *R. etli*.

In the rhizosphere, bacteria are exposed to the adverse effects that result from changes in salinity and temperature [25], thus affecting the survival of *Rhizobium* in the soil as well as the initial steps of symbiosis [25, 26]. Here, to further characterise the heat and salt shock responses, we explore the transcriptomic response of *R. etli* CE3 by RNA-Seq following heat (30 minutes at 42°C) and saline shocks (30 minutes in 80 mM NaCl). The first aim of this study was to identify genes that are differentially expressed under these stresses; additionally, we studied the ncRNAs and the promoter regions of the up-regulated genes. In doing so, we gained new insights into the global response of *R. etli* under both stress conditions. Our results indicate that replicon contribution is different for different stress responses; furthermore, it seems that the heat shock response is more complex compared to the saline shock response.

## Results

### Identifying the differentially expressed genes

To identify which genes are expressed following either heat shock or saline shock, nine RNA-Seq libraries were generated from *Rhizobium etli* CE3 growth in three different conditions: reference, heat shock and saline shock (see Methods). Three biological and independent experiments were performed for each condition; the general features of the results from each run are shown in Additional file 1. The libraries were sequenced and 15–50 million reads were obtained under the control condition, 16–53 million under heat shock and 16–53 million under saline shock. These numbers indicate that similar amounts of data were generated for the different conditions considered here. The Bowtie tool was used to map the reads to the annotated *R. etli* CFN42 genome (see Methods). An average of 4,877,844.67 (+/− 1,792,644.23) reads could be unambiguously mapped for the reference condition, 3,840,059 (+/− 1,208,900.91) for the heat shock condition, and 4,312,365.33 (+/− 2,340,976.94) for the saline shock condition (Additional file 1); as with the data generated, similar amounts of data could be mapped for each condition. Because these data were used in downstream analyses, we validated the data set by performing quantitative reverse transcription PCR (qRT-PCR) of twenty-seven genes selected from the RNA-Seq analysis (Additional file 2). We noted a high degree of correlation (R = 0.97) between the log2-transformed values of the quantification cycles (Cp) and the log2-transformed RPKM from the RNA-Seq data.

**Figure 1:**
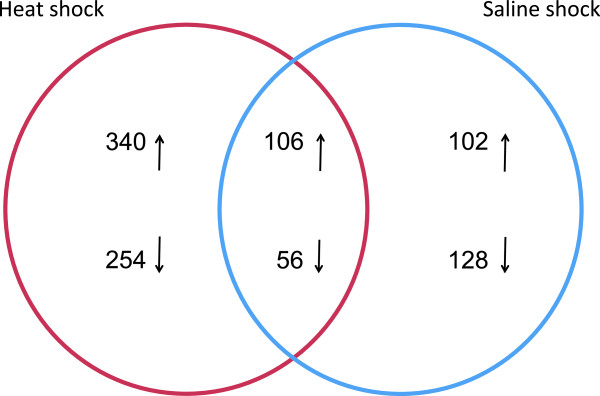
**Stress responses following heat and saline shock in** ***R. etli*** **.** Venn diagram showing how the over-expressed genes overlap for the heat shock and saline shock responses of *R. etli*. The arrows indicate the number of up-regulated and down-regulated genes in the conditions analysed. The intersection indicates the number of overlapping genes for both conditions.

**Figure 2:**
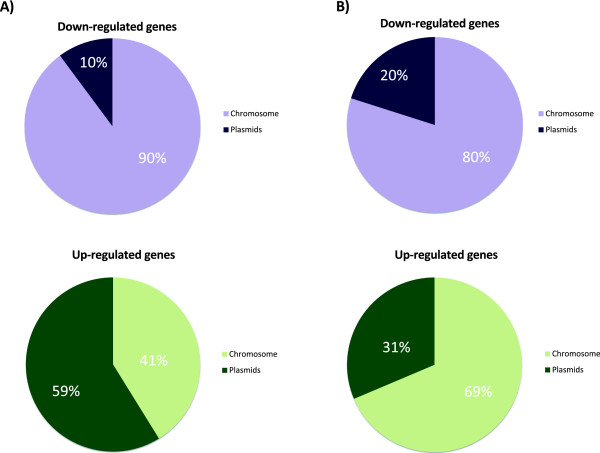
**Chromosomal and plasmid genes are differentially expressed following heat and saline shock.** Percentage of chromosomal and plasmid differentially expressed genes under **A)** heat shock and **B)** saline shock conditions.

**Figure 3:**
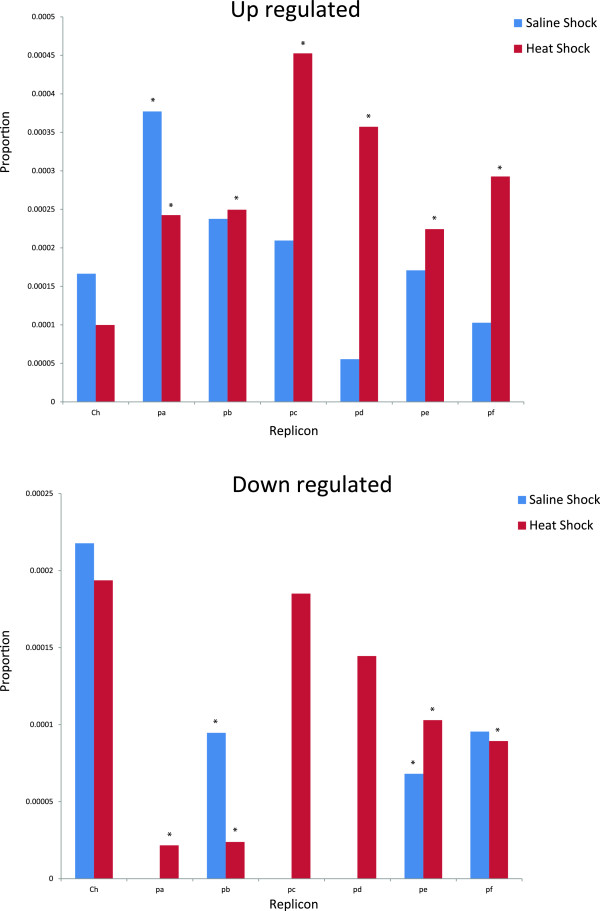
**Replicon distribution of over-expressed genes following heat and saline shock. Proportions of the up-regulated gene contributions for each replicon.** Axis labels correspond to: chromosome (Ch), plasmid pRet42a (pa), plasmid pRet42b (pb), plasmid pRet42c (pc), plasmid pRet42d (pd), plasmid pRet42e (pe), and plasmid pRet42f (pf). The proportions were obtained by normalising the number of up-regulated genes to the number of annotated genes for each replicon. *Indicates significant difference in the proportions of the up-regulated and down-regulated genes between the chromosome and plasmids (Two-sample for equality proportions with 99 percent confidence intervals).

A previous study showed that the *R. etli* genome has 5963 genes distributed across one chromosome and six large plasmids [20], to identify which of those are differentially expressed under each stress condition, a non-parametric approach was applied to the RNA-Seq data using NOISeq. The differential expression analysis revealed 756 differentially expressed genes (12.67% of the total) under heat shock conditions. Of these, 446 and 310 were up-regulated and down-regulated, respectively. Under saline shock conditions, we identified 392 (6.57%) differentially expressed genes. Of these, 208 and 184 were up-regulated and down-regulated, respectively. Among the total number of up-regulated genes, 340 were specific to heat shock, 102 were specific to saline shock and 106 were up-regulated under both conditions (Figure 1). Considering the total number of down-regulated genes, 254 were specific to heat shock, 128 were specific to saline shock and 56 were down-regulated under both conditions. These results suggest that the response to heat shock could be more complex because the number of differentially expressed genes following heat shock was twice the number observed following saline shock. To determine if these differentially expressed genes were homogeneously distributed across the different replicons, we analysed the location of these genes. We found that 59% of the genes over-expressed under heat shock conditions were located on plasmids, whereas 41% were located on the chromosome. In contrast, under saline shock conditions, 69% of the over-expressed genes were located on the chromosome, and 31% were found on plasmids. In the case of the down-regulated genes, 90% and 80% were chromosomally encoded under heat and saline shock conditions, respectively (Figure 2). Additionally, although we found that the over-expressed genes in the heat shock sample were principally located on all plasmids, this trend was more exacerbated in pRet42c, pRet42d, pRet42e and pRet42f, and the over-expressed genes in the saline shock sample were located on the chromosome and pRet42a. These observations suggest that replicon contribution is different under each stress response, where the pRet42c, pRet42d, pRet42e and pRet42f plasmids could be preferentially involved during the heat shock response, and the chromosome and pRet42a plasmid could play more significant roles during the saline shock response (Figure 3).

### Functions involved in the stress responses

**Figure 4:**
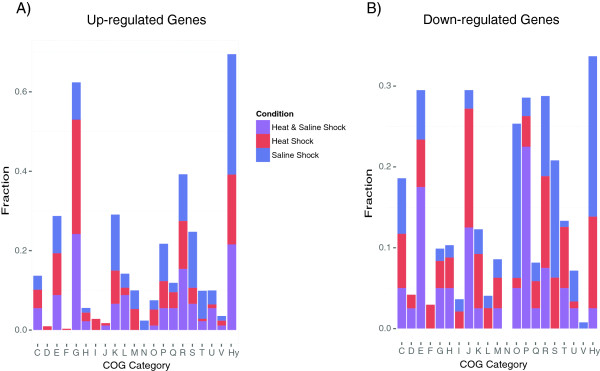
**Differentially expressed genes grouped by COG functional classification.** The fractions of the differentially expressed genes that fell within the various Clusters of Orthologous Gene (COG) categories. The columns are labelled as follows: C, energy production and conversion; D, cell division and chromosome partitioning; E, amino acid transport and metabolism; F, nucleotide transport and metabolism; G, carbohydrate transport and metabolism; H, coenzyme metabolism; I, lipid transport and metabolism; J, translation, ribosomal structure and biogenesis; K, transcription; L, DNA replication, recombination and repair; M, cell wall/membrane biogenesis; N, cell motility; O, posttranslational modification, protein turnover, chaperones; P, inorganic ion transport and metabolism; Q, secondary metabolite biosynthesis, transport and catabolism; R, general function prediction only; S, function unknown; T, signal transduction mechanisms; U, intracellular trafficking and secretion; V, defence mechanisms; and Hy, hypothetical proteins. Each fraction was obtained by normalising the number of total of genes assigned to each COG group per condition. **A)** Up-regulated genes. **B)** Down-regulated genes.

To evaluate which functions are intrinsic to the stress responses, the differentially expressed genes were grouped using the COG and KEGG databases. We successfully grouped 87% of the genes that were differentially expressed under heat shock, 80% of the genes under saline shock and 85% of the genes that were differentially expressed under both conditions; the rest of the genes could not be assigned to either the COG or the KEGG database. As expected, many of the differentially expressed genes were grouped into functional categories and pathways that are known to participate in adaptations to heat stress, saline stress and other stressful conditions (Figure 4). We next identified the specific genes that are up- or down-regulated under each condition separately and under both conditions.

### Chaperones act exclusively in the heat shock response

During the heat shock response, chaperones and proteases are involved in protein folding and the degradation of unfolded proteins, respectively [27–29]. Here, we found that *dnaK*, *grpE*, *groESch*_*2*_, *groEL*, *ibpA* and *clpB* were up-regulated in *R. etli* during heat shock stress. Of them, *dnaK* and the members of the chromosomally located *groESch2-groEL* operon showed the highest transcriptional levels. These observations were confirmed by qRT-PCR (Additional file 2). Other heat shock genes were over-expressed in our RNA-Seq data, including *groESf* and *groEL* from plasmid pRet42f and two members (*CH01244* and *CH01245*) of the HSP20 family of molecular chaperones. In total, fourteen heat shock proteins were over-expressed under heat shock conditions, whereas only two serine proteases (*degPch*_1_ and *degPch*_2_) were significantly over-expressed under saline shock conditions. Interestingly, no chaperones were found in the set of genes that was up-regulated under both conditions and numerous chaperones and proteases were down-regulated following saline shock (Additional file 3). These results could indicate that the recruitment of chaperone proteins seems to be exclusive to the heat shock response.

### Secondary metabolism is highly represented in the saline shock response

We found that *glpD*, *glgXe* and *PE00008* were over-expressed under saline shock conditions. The genes *glgX* and *PE00008*, which encode a glycosyl hydrolase (glycogen debranching) protein and a putative maltooligosyl trehalose synthase, respectively, may mediate glycogen accumulation during saline shock. According to the KEGG database, these genes are part of the *treYZ* pathway (Additional file 3), which is involved in converting maltodextrins (e.g., glycogen) into trehalose. Our results suggest that *R. etli* uses the *treYZ* pathway for the *de novo* synthesis of compatible solutes (i.e., carbohydrates and disaccharides, such as sucrose and trehalose) under saline stress conditions [30]. We also found that *ndvA*, which is required for cyclic glucan biosynthesis [31], was down-regulated following saline shock and up-regulated following heat shock. This is consistent with other studies that have shown a decrease in the expression levels of *ndvA* and *ndvB* when osmolarity increases in *Agrobacterium tumefaciens* [31, 32].

### Over-expression of secretion and transporter systems is a common response to heat and saline shock

Most bacterial genomes encode different secretion systems (such as toxin-antitoxin systems, pump efflux systems and ABC protein exporters), which provide versatile responses and adaptations to environmental changes. In our data, one heat shock-induced gene (*CH00527*) and three saline shock-induced genes (*CH1305*, *CH02813* and *PF00285*) were found to be members of the HlyD family, which is a type I secretion system. Furthermore, we also found that components of the type IV secretion system were up-regulated during both the heat and saline shock responses; these genes included *virB2a*, *PD00153* (*virB*_*11*_), *trbE* (under heat shock), *virD*_*2*_, *trbJ*, *trbD*, *trbB* and *PD00150* (VirB_8_); notably, under saline shock, the up-regulation of *trbI*, *trbG* and *trbF* was also triggered (Additional file 3). Under heat and saline shock conditions, the largest functional category of up-regulated genes corresponded to carbohydrate transport and metabolism (COG G). Of these up-regulated genes, 71% were ABC transporters. We also found that *R. etli* over-expressed an ABC-type transporter for spermidine (*CH03663)* under saline shock conditions; spermidine and spermine are polyamine derivatives relevant to various cellular events, such as cell differentiation and membrane functions [33, 34]. Therefore, we think that the large proportion of over-expressed ABC transporters suggests that these transporters could be playing an important role in bacterial cell viability by exporting secondary metabolites (polyamines), disaccharides and amino acids to contend with the stressful conditions.

### Versatile responses of transcription factors

Another functional category that was highly represented in *R. etli* during either the heat or saline stress responses was transcription (COG K). Twenty-six genes encoding transcriptional factors from the IclR, TetR, GntR and MarR families were up-regulated under heat shock conditions, while *CH00561*, *CH00796*, *CH03672* and *CH04029*, which encode transcriptional factors of the MarR, LysR and TetR families, were over-expressed under saline shock conditions. It is known that bacteria express different sigma factors under different environmental conditions. Of the 23 sigma factors present in *R. etli*, we observed over-expression of three of them following saline shock: *CH01118* (ECF subfamily), *rpoE*_*4*_ (σ^28^) and *rpoH*_2_ (σ^32^). The transcriptional factors identified here have been implicated in several processes [35–37], such as the osmotic shock and heat shock responses, among others (see Discussion), whereas one of the sigma factors, *rpoE*_*4*_, has also been implicated in the saline and osmotic stress responses [36].

### Promoter regions and ncRNAs

**Figure 5:**
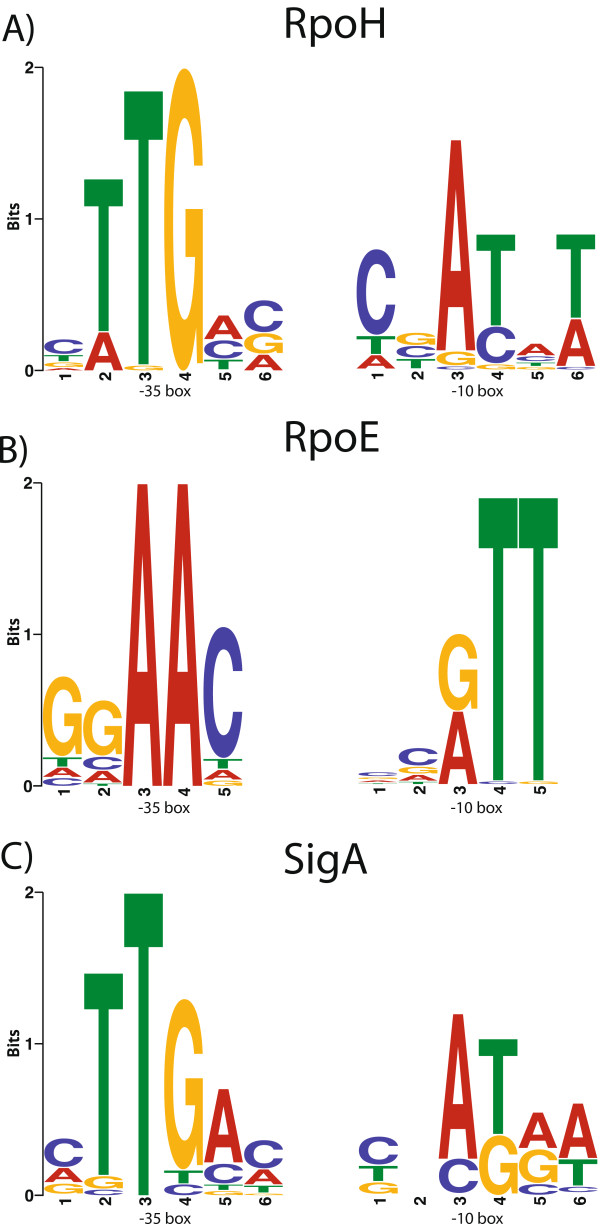
**Promoter consensus motifs of** ***R. etli*** **.** The sequence logos of three different sigma factors are shown. **A)** The logo shows the promoter consensus sequences for RpoH, as obtained from 107 putative promoters regions. **B)** The logo shows the promoter consensus sequences for RpoE, as obtained from 74 putative promoters regions. **C)** The logo shows the promoter consensus sequences for SigA, as obtained from 96 putative promoters regions (see Methods). The coloured sequence logos were generated using MEME. The scale of bits in each logo is represented by the height of each letter (nucleotide), showing the positional probability of that nucleotide multiplied by the information of the logo. The −35 box consensus motifs are displayed on the left side of the figure, and the −10 box consensus motifs are displayed on the right side of the figure. The 11 to 23-nt spacer region between the two boxes is not shown.

Thus far, we have considered the differentially expressed genes, but another important elements for the regulation of transcription are the promoter region and ncRNAs. First, to study the promoter regions in all of the non-coding regions associated with the genes over-expressed in both conditions, an *in silico* analysis to detect promoter motifs was conducted. Analysis of 266 up-regulated transcriptional units identified a ggAAC-N_16_-cgTT sequence and a cTTGAc-N_16_-cnATAA sequence in 147 transcriptional units (approximately 16 to 50 nt upstream relative to the ATG start codon), and this sequence perfectly matched the promoter consensus previously reported in *R. etli* for σ^28^ (RpoE_4_ and *PF00052*-ECF) and σ^70^ (SigA) [36, 38, 39]. An additional 105 motifs showed a cTTGaa-N_16_-CgATaT sequence, which is extremely similar to the *S. meliloti*’s σ^32^ (RpoH_1_ and RpoH_2_) reported motifs [40] (Figure 5). In our predictions, twenty-six putative promoters overlapped between RpoH and SigA, seven overlapped between RpoH and RpoE, six between RpoE and SigA and one among all three of these sigma factors (Additional file 4). Taken together, these results suggest that overlapping sigma promoter sequences could occur with high frequency and may be common in *R. etli*.

It is known that non-coding RNAs (ncRNAs) play an important role in bacterial gene expression; recent studies have identified ncRNAs in *R. etli*, and most of them are encoded in the intergenic regions [18]. Hence, to better understand the stress responses, we also conducted a screen to identify possible ncRNAs that are involved in these stress responses. We identified 107 intergenic regions of the *R. etli* genome that showed detectable and reproducible transcriptional activity (see Methods). Of the 107 regions, 89 had been previously reported as ncRNA candidates [18]. Eighteen regions (Table 1) revealed five (ReC106, ReC114, ReC115, ReC116 and ReC117) non-annotated proteins annotated in other genomes. Additionally, ReC103, ReC110, ReC112 and ReC113 matched hypothetical annotated proteins (YP_002547508.1, YP_001978587.1, WP_007533890.1 and WP_010007492.1, respectively) (Additional file 5). Among the remaining transcriptional units, ReC108 showed high levels of transcription, while ReC101, ReC102, ReC104, ReC105, ReC107, ReC118 and ReC111 showed low levels of transcription; these regions did not match any annotated proteins. Notably, ReC101 and ReC102 were located in the possible 5’-UTRs of their proximal genes, suggesting that they might be riboswitches. However, using Riboswitch finder and RiboSW (see Methods), these regions did not match any homologous riboswitches in other bacterial genomes; therefore, we considered them to be putative ncRNAs. We identified 13 differentially expressed ncRNAs under heat shock conditions and nine under saline shock conditions. Eight of the 13 differentially expressed ncRNAs were up-regulated following heat shock (ReCO6, ReC15, ReC20, ReC33, ReC55, ReC76, ReA01 and ReE02) and had been previously reported, including a well-characterised RNase P (ReC55). Under saline shock conditions, only two ncRNAs were over-expressed (ReC107 and ReC64); of them, one was a novel ncRNA identified in this study (ReC107; Additional file 6). Next, we used matrix predictions (see Methods) to identify potential promoter elements in the regions 80-nt upstream of the over-expressed ncRNAs. Most of the ncRNA upstream regions harboured motifs for more than one sigma factor, except for the novel ncRNA, ReC107, which yielded only a single putative SigA-dependent promoter. Rec06, Rec33 and Rec55 (RNase P) had putative RpoE-like and RpoH-like promoters while ReA01 and ReC64 harboured overlapping promoters for SigA and RpoH. The fact that we were able to identify potential motifs for sigma factors in the upstream regions of the ncRNAs provides additional evidence to support the notion that these ncRNAs are real. However, in order to learn about the possible function of these ncRNAs futher research is required; we started working in that direction (Gamaliel López-Leal *et al.*, in preparation). This analysis clearly indicates that ncRNAs are also playing a role in both the heat and saline shock stress responses.

**Table 1:** **Novel ncRNAs detected by RNA-Seq plotted data**

Id	Start	Stop	Strand	Length	Prediction
ReC 101	672255	672116	Minus	140 bp	ncRNA
ReC 102	1331946	1331792	Minus	155 bp	ncRNA
ReC 103	1674693	1674827	Plus	135 bp	Protein
ReC 104	1747454	1747598	Plus	145 bp	ncRNA
ReC 105	1748816	1748663	Minus	154 bp	ncRNA
ReC 106	1749213	1748896	Minus	318 bp	Protein
ReC 107	1832463	1832580	Plus	118 bp	ncRNA
ReC 108	1839511	1839637	Plus	127 bp	ncRNA
ReC 109	1932797	1932959	Plus	163 bp	ncRNA
ReC 110	2475589	2475733	Plus	145 bp	Protein
ReC 111	2546193	2546004	Minus	190 bp	ncRNA
ReC 112	2763999	2764196	Plus	198 bp	ncRNA
ReC 113	2940023	2939896	Minus	128 bp	ncRNA
ReC 114	3416634	3416858	Plus	225 bp	Protein
ReC 115	3620823	3620581	Minus	243 bp	Protein
ReC 116	3634855	3634540	Minus	316 bp	Protein
ReC 117	3719937	3719559	Minus	379 bp	Protein
ReC 118	3776471	3776654	Plus	184 bp	ncRNA

Table 1. Novel ncRNAs detected using the RNA-Seq plotted data. Eighteen transcribed regions were identified as novel ncRNAs or non-annotated proteins using Artemis (Graph, Add User Plot). The coordinates, strands and lengths of the ncRNAs and non-annotated proteins are shown (see the Methods section for more details).

## Discussion

The main goal of this study was not only to identify genes that are differentially expressed during the saline and heat shock responses but also to characterise other regulatory elements, namely ncRNAs and sigma promoter motifs. In doing so, we think a more comprehensive view of bacterial gene regulation and new insights into the global response of *R. etli* under both stressful conditions can be obtained.

In this study, we considered two stressful conditions, saline and heat shock. We found that these two responses in such conditions are clearly different from each other. First, it seems that transcriptional regulation of the heat shock response involves many more genes. Second, the replicon contribution is different during each stress response; pRet42c, pRet42d, pRet42e and pRet42f plasmids are preferentially involved in the heat shock response, whereas the chromosome and pRet42a plasmid participate in the saline shock response (Figure 3). Previous studies have also suggested the importance of plasmids for stress responses [41–43]. For instance, roles of plasmids (pRet42d and pRet42b) in nodule formation in *R. etli* and related species have been demonstrated [42]. Moreover, the contributions of pRet42a to pSym transfer and pRet42a, pRet42b and pRet42c to heat shock tolerance have been shown [43, 44]. Recent studies have shown that several bacteria have a clear genomic and transcriptional organisation [41, 45, 46]. For example, it was demonstrated *Caulobacter crescentus* has a chromosome that is spatially organised throughout its lifetime, and it was recently demonstrated that the naturally abundant groESL mRNA displayed a limited dispersion from its site of transcription during growth under normal and heat shock conditions. This result implies that the cell interior is functionally compartmentalised into small subcellular regions defined by the genetic map and organisation of the *C. crescentus* genome [45]. In *R. etli*, the chromosome and all of the plasmids encode active partition systems, which provide a specific location in the cell [47]. Taking this fact into account and considering the abundance of plasmid up-regulated genes following heat and saline shock, it is reasonable to suppose that plasmids are important mobile elements that provide a transcriptional spatial compartment to help organise the response to stress. Moreover, the abundance of up-regulated genes related with the stress response in *R. etli* plasmids could be an important adaptive advantage because sets of genes required for particular environmental stresses can be horizontally acquired and distributed across *R. etli* populations in a fast and efficient way.

Another clear feature that differentiates these two responses is the specific functions that are involved in each response. Next, we discuss the functions activated under each response and those activated in response to both stresses. Chaperones and proteases are involved in protein folding and the degradation of unfolded proteins during the heat shock response [27–29]. Along these lines, we found that *dnaK*, *grpE*, *groESch*_*2*_, *groEL*, *ibpA* and *clpB* were up-regulated under heat shock stress. Furthermore, three of the proteins encoded by these genes (DnaK, GrpE, GroESL) help to maintain the correct folding of nascent polypeptides under non-stressful conditions but become essential for survival under heat shock conditions. For instance, in *E. coli*, the association of DnaK, DnaJ and GrpE is crucial for the regulation of the levels of RpoH (σ^32^) [48–50], which is involved in controlling the gene expression of heat shock proteins. Notably, both *rpoH* mutants in *E. coli* [51, 52] and *rpoH*_*1*_ mutants in *R. etli* [37] are extremely sensitive to heat shock. Additionally, these heat shock proteins (*dnaK*, *groEL*, *groES* and *grpE*) have been also found over-expressed in *Rhizobium tropici*, which is recognized for its tolerance to high temperatures [53, 54]. Remarkably, while 14 heat shock proteins were over-expressed following heat shock, only two serine proteases *degPch*_1_ and *degPch*_2_ were significantly over-expressed under saline shock. These proteases were found to be involved in the elimination of inactive protein aggregates that accumulated in response to saline stress in *S. meliloti* [55–57]. Of note, the set of up-regulated genes shared in the two conditions did not include any chaperones; furthermore, numerous chaperones and proteases were down-regulated under saline shock condition (Additional file 3). Therefore, these results suggest that the recruitment of chaperone proteins seems to be exclusive to the heat shock response.

In Rhizobia, glycogen accumulation helps restore cell volume after osmotic shock [29], and high expression levels of *glgA2*, *glgB2* and *glgX* in *S. meliloti* support this conclusion [30]. A study in *Rhizobium tropici* PRF81 has reported that *glgX* together with nod-genes could be involved in the signal exchanges between host plant and rhizobia [58]. Here, we noted that *glpD*, *glgXe* and *PE00008* were over-expressed in *R. etli* under saline shock. These genes are part of the *treYZ* pathway, which converts maltodextrins (e.g., glycogen) into trehalose, thus suggesting that *R. etli* uses the *treYZ* pathway for the *de novo* synthesis of compatible solutes (i.e., carbohydrates and disaccharides, such as sucrose and trehalose) under saline stress conditions [30]. We think this might be true as trehalose and glycine betaine are known to function as osmotic stabilisers [59], and trehalose biosynthesis is a common response to osmotic stress in many bacteria [30, 60]. All this information suggests that these genes have an important roll not only in the free-living state during stressful conditions, but also in the symbiotic state with the host-plant *P. vulgaris*. Furthermore, in *Rhizobium*, the accumulation of betaine and trehalose allows the bacterium to survive under osmotic stress conditions and reduces the negative effects of NaCl [61].

Our data suggest that the over-expression of secretion and transporter systems may be a common response to both heat and saline shock responses. For instance, *CH00527* and *CH1305*, *CH02813, PF00285* were found to be members of a type I secretion system (HlyD family), which exports a variety of compounds, from drug molecules to large polypeptides, and is mainly involved in the secretion of noxious molecules across the cellular envelope [62–64]. Recent studies have suggested that type I secretion systems are involved in the translocation of unfolded proteins [65–68]. Additionally, a set of genes (*virB2a*, *PD00153* [*virB*_*11*_], *virD*_*2*_, *trbJ*, *trbD*, *trbB* and *PD00150* [VirB_8_]) from the type IV secretion system was up-regulated during both the heat and saline shock responses. Of note, under saline shock conditions, the up-regulation of *trbI*, *trbG* and *trbF* is also triggered, whereas *trbE* is over-expressed following heat shock. Proteins from the type IV secretion system participate in a system that mediates the conjugative transfer of DNA with the extracellular milieu, and some even deliver DNA to fungi, plants or human cells [63, 64]. Obviously, this process plays an important role in bacterial fitness under changing environmental conditions. Noticeably, under heat and saline shock conditions, the largest functional category of up-regulated genes corresponded to carbohydrate transport and metabolism (COG G) - most of these genes encode ABC transporters. These proteins belong to one of the two mayor groups of transporters indentified in gram-negative bacteria, which can carry a wide variety of substrates, including sugars, amino acids, polysaccharides and peptides [69–71]. This trend does not seem to be exclusive to *R. etli,* as the genomes of α-proteobacteria tend to encode disproportionately large numbers of ABC transporters, reflecting their ecological versatility and their need to adapt to various conditions [8]. Following heat and saline shock, one-third of the up-regulated genes encode ABC transporters; of these genes, 18.5% were annotated as sugar transporters. In *S. meliloti* and other rhizobia, disaccharides have also been recognised as osmoprotectants [72]. We also found that ABC-type transporter for spermidine (*CH03663)* that was over-expressed following saline shock. Polyamines have been shown to be crucial for salt tolerance and the osmotic stress responses of soil microorganisms [73], as they can act as osmoprotectants because their cationic nature allows them to bind to proteins and lipids and thus stabilise cellular structures [74]. Additionally, we noted repression of the *CH03629* gene under both conditions, which encodes an ornithine decarboxylase responsible for decarboxylating ornithine to putrescine, suggesting that at 30 min after induced stress, the synthesis of spermidine could be repressed. Therefore, we think that many of the over-expressed ABC transporters play an important role in cell viability by exporting secondary metabolites (polyamines), disaccharides and amino acids to contend with the stressful conditions. Besides, these ABC transporters could be part of the general stress response in *R. etli*.

Sigma factors are key to the stress response, as the use of alternative sigma factors creates flexibility in bacterial adaptation. Here, we observed over-expression of three sigma factors following saline shock: *CH01118* (ECF subfamily), *rpoE*_*4*_ (σ^28^) and *rpoH*_2_ (σ^32^). In *R. etli,* RpoE_4_ participates under oxidative, saline and osmotic stresses and also controls the expression of *rpoH*_2_, whereas *rpoH*_2_ mutants were reported to be sensitive to NaCl shock [36, 37]. Thus, these two genes play roles in cell survival during the saline shock response. We also detected constitutive expression of *rpoH*_1_ under heat shock and in the reference condition, which was previously shown to be essential for the heat shock response in *R. etli* [37]. Similar patterns have been observed in other bacteria, where the levels of mRNA-*rpoH* are maintained at a basal concentration [75]. In *E. coli*, the mRNA of *rpoH* contains a thermo-switch within the 5’-UTR of the mRNA [76] that under non-stressful conditions blocks the translation of the *rpoH* gene, whereas under stressful conditions (e.g., heat shock) allows the translation of *rpoH* [76]. Studies in *B. japonicum* have demonstrated that this level of regulation is also present in rhizobial species [77]. For example, some heat shock genes are translationally controlled by a secondary mRNA structure called ROSE (repression of heat shock gene expression); this regulatory element mainly affects genes that encode small heat shock proteins, but it also controls *rpoH*_1_, which encodes one of the three σ^32^ factors in *B. japonicum* [77]. Future studies are warranted to investigate whether an analogous mechanism exists in *R. etli*. Additionally, the regulatory region of *rpoH*_1_ in *R. etli* contains two promoters: one for σ^70^ and one for σ^28^ [37]. These results indicate that the constitutive expression of *rpoH*_1_ in *R. etli* could be regulated by its primary sigma factor (σ^70^). However, the qRT-PCR expression data revealed that *CH01118* is differentially expressed under heat shock, in contrast with the observed data in the RNA-Seq analyses. These results could be explained by the fact that in the qRT-PCR experiments, the cDNAs were obtained with specific primers, ensuring that the cDNA belonged to the target gene. Additionally or alternatively, the biological noise in our RNA-Seq libraries could be the reason for this (see Methods). Nonetheless, the regulation of RpoH by RpoE is retained in many bacteria. In this study, we identified the over-expression of three sigma factors; two of them belong to sigma factor RpoE family, which could regulate the RpoH sigma factors in *R. etli* under heat and saline shock. However, further studies on the 18 ECF-sigma factors and their targets should be performed in *R. etli* because the regulatory networks of these sigma factors have not been fully explored.

The identified promoter motifs were expected as the transcriptomes were obtained under stressful conditions, where both RpoH_1_ and RpoH_2_ are essential. Clearly, this approach does not discriminate promoter motifs between the alternative sigma factors RpoH_1_ - RpoH_2_ and RpoE_4_ - *CH01118-*ECF. However, studies of the *R. etli* regulons RpoE_4_ and *PF00052*-ECF have identified a unique sequence motif, which has been recognised for both ECF-type sigma factors, resulting in a large number of overlapping regulated genes [35]. Moreover, analysis of RpoH’s regulons in *S. meliloti* has identified the same tendency of a high number of similar sequences in their promoters [40]. A recently massive exploration and mapping of σ^H^, σ^E^ and σ^70^ promoters in *S. meliloti* revealed highly similar promoter sequences between alternative sigma factor copies [78]. These results indicated that the regulation of the different sigma factors in a given genome could depend on alternative and more complex regulatory systems that include the participation of transcriptional factors, riboswitches, ncRNAs and posttranslational regulatory strategies. Therefore, specific sequence-promoter recognition may be limited to a few events. In support of this idea, it is well known that in *E. coli* and other bacterial genomes, promoters belonging to the σ^70^ family can overlap [35, 52, 79, 80]. For instance, in the case of *E. coli,* overlapping RpoH and RpoD (σ^70^) promoters have also been reported [81]. Here, we note that in the overlapped promoters, the −35 box was maintained in the two promoter predictions, and only the −10 box was displaced. In most cases, this displacement involved only a few bases in either direction (upstream or downstream). This result is also supported by the observation of lax promoter recognition in *R. etli* (principally in the −10 box) [39] and by the occurrence of additional promoter-like signals that could play a regulatory role. In bacteria, the promoter-like signals tend to be generated easily, resulting in selection for functionally redundant promoter sequences [82]. Alternatively, the complex regulation of the −10 box has led several authors to consider that it may comprise a different type of promoter [2]. In *E. coli*, the −10 box (unlike the −35 box) is not a single recognition element; instead, it seems to function as two recognition elements: one primarily positioned at −12 in the double strand and another based on the melting of the −11 to −7 positions in a single strand [2]. Our results, along with the findings from other studies, suggest that the overlapping of motifs for the different sigma factors might be a common occurrence, which in turn suggests that additional elements (riboswitches, ncRNAs and post-transcriptional regulatory elements) could be playing roles in determining the specificity of any given stress response.

The regulatory networks that allow bacteria to respond to different environmental stresses usually comprise transcriptional regulators, sigma factors, proteases and small ncRNAs [18]. In this work, we identified 13 differentially expressed ncRNAs under heat shock and 9 under saline shock conditions. Of note, this result is consistent with our finding that more genes are up-regulated during the heat shock stress response, as here we also find that more ncRNAs were up-regulated during that stress response. We think these ncRNAs are real entities for several reasons. First, some of them have been previously reported. In the case of the heat shock response, eight of the up-regulated ncRNAs (ReCO6, ReC15, ReC20, ReC33, ReC55, ReC76, ReA01 and ReE02) were previously described [18, 83], whereas under saline shock conditions, two ncRNAs (ReC107 and ReC64) were identified previously [18]. Secondly, we detected reproducible transcriptional activity (in biological replicates); besides, sigma promoter motifs were identified for some of these ncRNAs. The analysis on ncRNAs in this work show that these molecules are an important part of the response to both heat and saline shock stress, future studies are required to establish relevance of these molecules in such stresses.

In considering the results, we believe that the heat shock response is more complex than the saline shock response. Not only does the heat shock response involve more differentially expressed genes and more ncRNAs, it also uses more replicons. Further studies are required to establish if these observations also occur at the translational level.

## Conclusions

We think our work exemplifies how the utilisation of approaches that consider more than just the differentially expressed genes and also include regulatory elements of the stress response provide a more comprehensive view of bacterial gene regulation. More specifically, this study gives new insights into the global response of *R. etli* under both stressful conditions.

## Methods

### Culture conditions

Nine independent cultures of wild-type *Rhizobium etli* CE3 [84] were grown at 30°C with agitation (200 rpm) in peptone-yeast extract (PY) medium [85]. When required, nalidixic acid (20 μg ml^−1^) was added to the cultures. Aliquots (10 ml) were taken from all of the cultures at 12 hours post-inoculation (OD_600_ ≈ 0.4, exponential phase) and incubated for 30 min at 30°C in PY medium (reference condition), at 42°C in PY medium (heat shock condition) or at 30°C in PY medium containing 80 mM NaCl (saline shock condition).

### RNA extraction and sequencing

Each sample was centrifuged at 13,000 rpm for 10 min, and the pellet was washed four times with 1 ml of Tris:EDTA buffer 50/20 (pH 8.0) and 500 μl of RNAlater solution (Ambion). Total RNA was isolated using an RNeasy Midi Kit (Qiagen) according to the manufacturer’s instructions. We verified the amount and quality of the resulting total RNA samples by agarose gel electrophoresis, as previously reported [86].

Ribosomal RNA was eliminated using a RiboMinus Kit (Life Technologies), which was used according to a modified version of the manufacturer’s recommended protocol. Briefly, a second rRNA elimination step using the Terminator Exonuclease enzyme (Epicenter), which uses RNA monophosphate as a substrate while leaving mRNA intact, was included in the protocol. RNA integrity was assessed using an Agilent Bioanalyzer 2100 and an RNA Nano 6000 Labchip Kit (Agilent Technologies). RNA sequencing was carried out by the Unidad Universitaria de Secuenciación Masiva de DNA (UUSMD)–UNAM (http://www.uusmd.unam.mx/), using a Next Generation High Throughput Sequencing Genome Analyzer IIx (GAllx; Illumina).

### Quantitative reverse transcription PCR

Quantitative reverse transcription PCR (qRT-PCR) of twenty-seven selected genes was performed. These selected genes represented differentially and non-differentially expressed genes under heat and saline shock conditions observed by RNA-Seq analysis. Total RNA was obtained from nine independent cultures (representing the nine RNA-Seq libraries) using an RNeasy Midi Kit (Qiagen). Total RNA (DNA free) was reverse transcribed into cDNA using the RevertAid H minus First Strand cDNA Synthesis kit (Thermo Scientific). Quantitative PCR was performed using a PCR system 3700 (Applied Biosystems). The expression levels of target genes were normalised to the expression level of the reference gene *hisCd*. The fold changes of three biological and technical replicates for each condition were obtained using the ∆∆C_t_ method [87]. The detected genes and primers are listed in Additional file 7.

### Mapping and analysis of RNA-Seq data

RNA-Seq data were aligned using Bowtie (http://bowtie-bio.sourceforge.net), with the *R. etli* CFN42 genome (ftp://ftp.ncbi.nlm.nih.gov/genomes/Bacteria/Rhizobium_etli_CFN_42_uid58377/) as the reference genome. Our mapping parameters were set to align the best quality reads (−−phred64-quals), report the best alignment and allow zero mismatches. To adjust the values in the biological replicates, the RNA-Seq data were pre-processed. The raw count expression profiles were pre-processed by the total number of reads from the library for each condition. This type of adjustment has also been performed by other packages [88].

A Perl script was written to determine the expression value of each gene, which used the output files of Bowtie. To visualise our RNA-Seq expression data, we used the output files of MAQ aligner (http://maq.sourceforge.net/maq-man.shtml); the quality parameter used in the MAQ pileup was q30, and zero mismatches were allowed. We built plots using the solexa2plots.pl script, which was generated based on the instructions in [89]. The output files could then be read into the Artemis software (http://www.sanger.ac.uk/resources/software/artemis/).

### Identification of novel ncRNAs and ORFs

To identify novel ncRNAs and non-annotated ORFs, we used the plots visualised in Artemis (see above) to manually identify the transcribed non-coding regions. The transcribed non-coding regions were then checked for folding using the RNA fold tool (http://rna.tbi.univie.ac.at/cgi-bin/RNAfold.cgi) and assessed to identify which strand could be transcribed. Basic Local Alignment Search Tool (BLAST; http://blast.ncbi.nlm.nih.gov) searches were used to verify that the regions did not match with proteins annotated in other genomes. The parameters used to identify non-annotated proteins were as follows: > 90% coverage of the subject protein and > 30% identity. We used this approach in conjunction with searches of the Rfam (http://rfam.sanger.ac.uk/) and miRBase (http://www.mirbase.org/) databases.

### Assessment of differentially expressed genes

To reduce the noise between the RNA-Seq replicates caused by differences in sequencing depth, we pre-processed the raw-count data by dividing them with the total number of reads from the library. We obtained Pearson’s correlation coefficients of 0.75, 0.74 and 0.75 for between-replicates for the reference, heat and saline shock conditions, respectively. These results are consistent with previous reports [18]. Because our biological replicates of the RNA-Seq data had different sequencing depths, we used the NOISeq package (http://bioinfo.cipf.es/noiseq/) to detect differential expression. We performed our analysis according to the provided instructions. We normalised our pre-processed data using the upper quartile method [18, 88–92] and applied k = 0.5 as recommended for normalised data in the NOISeq manual.

### Functional categorisation of genes

We used the Clusters of Orthologous Groups (COG; http://www.ncbi.nlm.nih.gov/COG/) and KEGG Automatic Annotation Server (KAAS; http://www.genome.jp/tools/kaas/; http://www.genome.jp/kegg/) databases to cluster the differentially expressed genes into functional categories and assign them to metabolic pathways.

### Consensus motifs

To identify promoter-like signals, we used the RSA tools (http://rsat.ulb.ac.be/). We built matrices for three sigma factors (RpoH, RpoE, and SigA) using the reported consensus sequences of SigA and RpoE in *R. etli* [36, 39] and those of RpoH and SigA in *S. meliloti* [38, 40]. We identified genes that were up-regulated in response to the two stress conditions and searched the 80 nucleotides (nt) upstream of their start codons for promoter-like signals. In cases where the transcriptional profiles appeared to start > 80 nucleotides upstream from the start codon, we used our RNA-Seq data plots to identify the potential promoter. To search for the sigma-factor-binding motifs, we used the matrix-scan tool from RSA tools (http://embnet.ccg.unam.mx/rsa-tools/). For our analysis of logo consensus sequences, we used the RSA tools consensus service and the Multiple Em for Motif Elicitation program (http://meme.nbcr.net). The identified promoters are listed in Additional file 8.

### RNA-Seq data accession number

The RNA-Seq sequence data have been deposited in the GEO (Gene Expression Omnibus) database under the accession number GSE50018.

## Authors’ information

This research was conducted by GLL in partial fulfilment of the requirements for a Ph.D. in Ciencias Biomédicas from the UNAM, Cuernavaca, México.

## Electronic supplementary material

Additional file 1: **General features of the total sequenced and mapped reads.** General features of the total sequenced and mapped reads. The reads were mapped using Bowtie aligner with zero mismatch criteria and the best quality reads (−−phred64-quals). See the Methods section for more details. (DOCX 65 KB)

Additional file 2: **qRT-PCR fold changes of twenty-seven selected genes observed by RNA-Seq.** Fold difference values were calculated using the ∆∆C_t_ method and normalised to the reference gene *hisCd* for 27 selected genes with differential and non-differential expression observed in the RNA-Seq data. Fold change values were obtained as an average over three biological and technical replicates. (DOCX 168 KB)

Additional file 3: **The Gen ID and COG designations of the genes that were up- and down-regulated in** ***R. etli*** **under heat and saline shock conditions.** (XLSX 82 KB)

Additional file 4: **Sigma-factor overlap among the promoters of genes that were up-regulated under heat and saline shock conditions.** Proportional Venn diagram of promoters with sigma-factor overlap for the genes that were up-regulated under heat and saline shock conditions. Independent of the tested conditions, 71, 62 and 60 promoters appeared to be exclusive to RpoH, SigA and RpoE, respectively, while twenty-six promoters overlapped between RpoH and SigA, seven overlapped between RpoH and RpoE, six between SigA and RpoE, and one across all three sigma factors. (PDF 97 KB)

Additional file 5: **BLAST analysis of ncRNAs and non-annotated proteins.** (XLSX 21 KB)

Additional file 6: **Differential expression of novel ncRNAs.** (XLSX 10 KB)

Additional file 7: **Gene and primer sequences for qRT-PCR analysis.** (XLSX 13 KB)

Additional file 8: **Putative sigma factor dependent promoters.** (XLSX 36 KB)
