# Supplementary material for: RNA-Seq analysis of the multipartite genome of Rhizobium etli CE3 shows different replicon contributions under heat and saline shock
Source: BMC Genomics. 2014 Sep 8;15(1):770. doi: 10.1186/1471-2164-15-770 (PMC4167512; doi:10.1186/1471-2164-15-770)
Supplement: Supplementary file 1 — Additional file 1: General features of the total sequenced and mapped reads. General features of the total sequenced and mapped reads. The reads were mapped using Bowtie aligner with zero mismatch criteria and the best quality reads (−−phred64-quals). See the Methods section for more details. (DOCX 65 KB) [file 12864_2014_6445_MOESM1_ESM.docx]

General features of the total sequenced and mapped reads.

| **Sample condition** | **Total No. of reads** | **Total No. of reads mapped + Bowtie** |
| --- | --- | --- |
| Control 1 | 15 603 608 | 3 116 800 |
| Control 2 | 29 782 478 | 4 816 234 |
| Control 3 | 50 329 512 | 6 700 500 |
| Heat Shock 1 | 16 694 238 | 3 180 493 |
| Heat Shock 2 | 17 563 245 | 3 104 398 |
| Heat Shock 3 | 52 417 613 | 5 235 286 |
| Saline Shock 1 | 16 399 769 | 4 143 526 |
| Saline Shock 2 | 15 958 635 | 2 060 379 |
| Saline Shock 3 | 52 675 237 | 6 733 191 |

General features of the total sequenced and mapped reads. The reads were mapped using Bowtie aligner with zero mismatch criteria and the best quality reads (--phred64-quals). See the Methods section for more details.
