# Supplementary material for: RNA-Seq analysis of the multipartite genome of Rhizobium etli CE3 shows different replicon contributions under heat and saline shock
Source: BMC Genomics. 2014 Sep 8;15(1):770. doi: 10.1186/1471-2164-15-770 (PMC4167512; doi:10.1186/1471-2164-15-770)
Supplement: Supplementary file 2 — Additional file 2: qRT-PCR fold changes of twenty-seven selected genes observed by RNA-Seq. Fold difference values were calculated using the ∆∆Ct method and normalised to the reference gene hisCd for 27 selected genes with differential and non-differential expression observed in the RNA-Seq data. Fold change values were obtained as an average over three biological and technical replicates. (DOCX 168 KB) [file 12864_2014_6445_MOESM2_ESM.docx]

**qRT-PCR validation of RNA-Seq data.**

| **Gene id** | **Fold difference in reference condition relative to heat shock** | **Fold difference in reference condition relative to saline shock** | ***P*-value heat shock** | ***P*-value saline shock** | **Gene function** |
| --- | --- | --- | --- | --- | --- |
| *CH00233* | 1 (0.84 – 1.18) | 1 (0.87 – 1.14) | 0.085 | 0.035 | Histidinol phosphate aminotransferase protein |
| *CH00073*^H^ | 1.8 (1.60 – 2.05) | 0.1 (0.08 – 0.12) | 0.610 | 0.220 | Sugar ABC transporter |
| *CH00084*^H*^ | 0.99 (0.87 – 1.12) | 0.33 (0.29 – 0.37) | 0.528 | 0.360 | Sugar ABC transporter, permease protein |
| *CH00145*^H^ | 1.82 (1.56 – 2.13) | 0.69 (0.60 – 0.79) | 0.558 | 0.736 | Molecular chaperone DnaK |
| *CH00526^H^* | 2.01 (1.72 – 2.35) | 0.16 (0.15 – 0.18) | 0.907 | 0.257 | TetR family transcriptional regulator |
| *CH01239*^H^ | 11.75 (8.07 – 17.12) | 0.09 (0.08 – 0.1) | 0.889 | 0.650 | Chaperonin GroEL |
| *CH03571*^H^ | 1.51 (1.15 - 1.99) | 0.4 (0.36 – 0.45) | 0.526 | 0.823 | Molecular chaperone small heat shock protein |
| *CH03753*^H^ | 3.59 (2.7 – 4.6) | 0.78 (0.65 – 0.93) | 0.764 | 0.665 | ATP-dependent Clp protease |
| *CH04007*^H^ | 0.27 (0.21 – 0.34) | 0.23 (0.21 – 0.26) | 0.519 | 0.308 | DeoR family transcriptional regulator |
| *CH01118*^S*^ | 5.05 (4.46 – 5.71) | 0.21 (0.19 – 0.24) | 0.420 | 0.548 | ECF subfamily RNA polymerase sigma factor |
| *CH01120*^S^ | 1.69 (1.42 – 2.00) | 1.76 (1.48 – 2.09) | 0.466 | 0.630 | Serine protease DO-like protein |
| *CH01293*^S^ | 0.84 (0.73 – 0.95) | 7.17 (6.38 – 8.04) | 0.270 | 0.778 | Serine protease DO-like protein |
| *CH02971*^S*^ | 1.72 (1.51 – 1.97) | 0.2 (0.17 – 0.23) | 0.034 | 0.605 | C4-dicarboxylate transporter DctA |
| *CH03663*^S^ | 2.26 (1.83 – 2.81) | 1.28 (1.15 – 1.43) | 0.425 | 0.519 | Spermidine/putrescine ABC transporter |
| *CH04026*^S*^ | 0.31 (0.27 – 0.36) | 0.57 (0.51 – 0.63) | 0.158 | 0.610 | RNA polymerase sigma factor *rpoH*_2_ |
| *CH00746*^N^ | 0.48 (0.41 – 0.55) | 0.28 (0.25 – 0.32) | 0.030 | 0.113 | RNA polymerase sigma factor *rpoE*_1_ |
| *CH03273*^S^ | 0.79 (0.69 – 0.91) | 1.12 (0.99 – 1.26) | 0.017 | 0.466 | RNA polymerase sigma factor *rpoE*_4_ |
| *CH03603*^N^ | 0.28 (0.22 – 0.34) | 0.04 (0.03 – 0.04) | 0.464 | 0.178 | Sarcosine oxidase beta subunit protein |
| *CH04054*^N^ | 0.43 (0.32 – 0.57) | 0.03 (0.03 – 0.04) | 0.178 | 0.207 | Oxidoreductase |
| *CH04095*^S*^ | 0.17 (0.15 – 0.20) | 0.03 (0.03 – 0.04) | 0.580 | 0.527 | Transporter, permease |
| *CH00460*^H^ | 3.4 (2.9 – 3.8) | 0.21 (0.18 – 0.24) | 0.868 | 0.439 | Zinc-binding oxidoreductase |
| *CH00465*^B*^ | 1.34 (1.16 – 1.54) | 0.28 (0.24 – 0.32) | 0.878 | 0.898 | frcB; fructose ABC transporter |
| *CH00527*^H^ | 48.72 (41.7 – 56.93) | 0.22 (0.19 – 0.26) | 0.959 | 0.136 | HlyD family protein secretion protein |
| *CH01330*^N^ | 0.43 (0.37 – 0.49) | 0.17 (0.15 – 0.20) | 0.109 | 0.465 | Glyoxalase protein |
| *CH01508*^N^ | 0.22 (0.19 – 0.25) | 0.13 (0.11 – 0.16) | 0.397 | 0.182 | glyA; serine hydroxymethyltransferase |
| *PF00229*^H*^ | 0.39 (0.34 – 0.44) | 0.42 (0.37 – 0.47) | 0.534 | 0.482 | Isomerase |
| *PF00565*^H^ | 1.2 (1.04 – 1.39) | 0.05 (0.04 – 0.05) | 0.519 | 0.370 | Sugar ABC transporter |

The normalized expression value by ∆∆C_t_ method, with the expression of reference gene

*CH00233* the standard deviation show in parentheses over three biological samples. The last two columns show the *P*-value obtained by NOISEq package.

^H^ Up-regulated genes under heat shock according to RNA-Seq analysis.

^S^ Up-regulated genes under saline shock according to RNA-Seq analysis.

^B^ Up-regulated genes under both conditions according to RNA-Seq analysis.

^N^ Non-differential expressed genes in the study according to RNA-Seq analysis.

* Indicate those genes that were differential expressed (heat and saline shock) by NOISEq and have no significance difference in expression value by qRT-PCR
