# Supplementary figures and images for: RNA-Seq analysis of the multipartite genome of Rhizobium etli CE3 shows different replicon contributions under heat and saline shock
Source: BMC Genomics. 2014 Sep 8;15(1):770. doi: 10.1186/1471-2164-15-770 (PMC4167512; doi:10.1186/1471-2164-15-770)

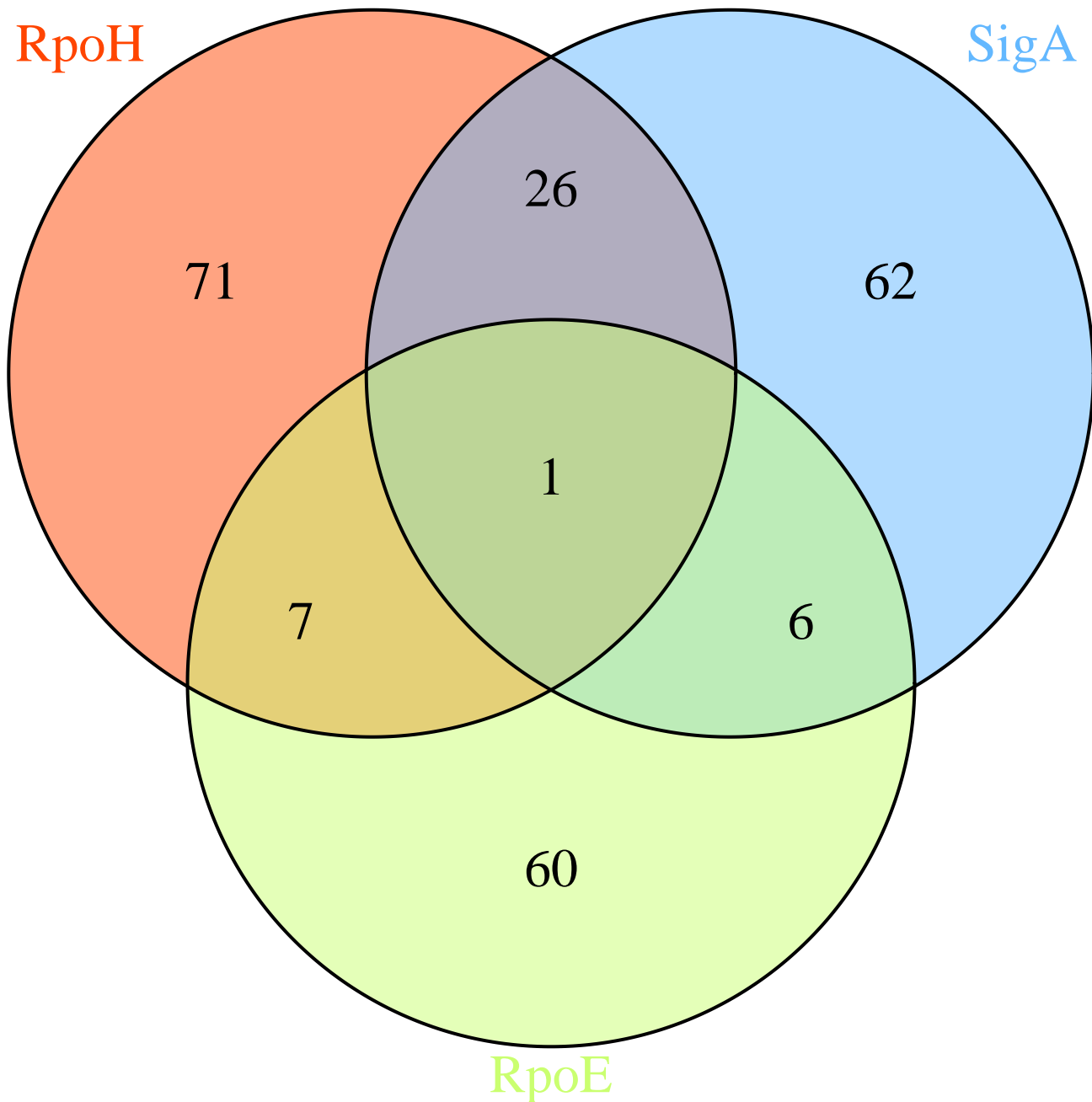

Supplement: Supplementary file 4 — Additional file 4: Sigma-factor overlap among the promoters of genes that were up-regulated under heat and saline shock conditions. Proportional Venn diagram of promoters with sigma-factor overlap for the genes that were up-regulated under heat and saline shock conditions. Independent of the tested conditions, 71, 62 and 60 promoters appeared to be exclusive to RpoH, SigA and RpoE, respectively, while twenty-six promoters overlapped between RpoH and SigA, seven overlapped between RpoH and RpoE, six between SigA and RpoE, and one across all three sigma factors. (PDF 97 KB) [file 12864_2014_6445_MOESM4_ESM.pdf]
